# Discovery of a defence-response gene for *Eldana saccharina* resistance in sugarcane

**DOI:** 10.3389/fpls.2026.1876002

**Published:** 2026-08-14

**Authors:** Robyn Moira Jacob, Meenu Ghai, Shailesh Vinay Joshi

**Affiliations:** 1 South African Sugarcane Research Institute, Durban, South Africa; 2 School of Agriculture and Science, College of Agriculture, Engineering and Science, University of KwaZulu-Natal, Durban, South Africa

**Keywords:** *Eldana saccharina*, insect resistance, marker assisted breeding, PAV, RNA-seq, sugarcane

## Abstract

*Eldana saccharina* is a major stalk boring pest of sugarcane in South Africa, causing substantial yield and revenue losses. Developing sugarcane cultivars with durable resistance to *E. saccharina* is therefore a priority for breeding programmes. In this study, we employed RNA sequencing (RNA-seq) to characterise the transcriptional responses to *E. saccharina* by comparing infested plants with uninfested controls in sugarcane genotypes displaying contrasting resistance phenotypes. Differential expression analysis, combined with functional annotation, revealed clear and contrasting defence responses between resistant and susceptible genotypes. Distinct sets of co-regulated genes were identified, reflecting genotype-specific immune activation patterns. The detection of both early and late transcriptional responses highlights how the timing and coordination of gene expression contribute to resistance or susceptibility outcomes. Integrating these expression signatures identified a candidate region harbouring a gene that was strongly induced in resistant genotypes following infestation. This genomic region exhibited presence/absence variation (PAV) among the genotypes analysed, enabling the development of a robust PCR-based marker for reliable tracking of the locus across diverse germplasm. The marker was subsequently assessed by screening genotypes from a late-stage breeding trial. The analysis revealed an association between the locus presence and enhanced resistance to *E. saccharina*. Collectively, these findings provide novel molecular insight into the sugarcane-*E. saccharina* interaction and identify a resistance-associated PAV locus that can be deployed as a practical PCR-based defence-response marker. This marker offers a useful tool to accelerate selection of resistant genotypes through marker-assisted breeding.

## Introduction

Agricultural productivity is severely constrained by insect pests which inflict extensive damage to crops, resulting in significant yield losses and posing a serious threat to global food security. It has been estimated that yield losses attributed to insect pests in major staple crops such as rice, maize, and wheat increase by approximately 10 to 25% for every degree of global mean surface warming (Deutsch et al., 2018). Conventional pest management strategies rely heavily on chemical pesticides, and global pesticide use has increased by approximately 14% over the past decade (FAO, 2025), largely driven by the need to meet the growing food demand. However, intensive pesticide use poses significant environmental challenges, including contamination of soil and water resources, negative impact on non-target organisms, and disruption of ecological balance. Prolonged and excessive application can also contribute to biodiversity loss and the evolution of pesticide resistant pest populations, raising concerns about the long-term sustainability of pesticide-dependent agricultural systems.

In response to these challenges, Integrated Pest Management (IPM) has emerged as a more sustainable approach to pest control. IPM integrates multiple complementary strategies, including biological control, cultural practices, physical methods, and the use of resistant crop varieties to reduce reliance on chemical pesticides. Among these strategies, the development of pest-resistant crop cultivars plays a critical role in sustainable pest management as it enhances crop productivity whilst simultaneously reducing reliance on insecticides and associated environmental and economic costs (Brummer et al., 2011).

Sugarcane (*Saccharum* spp.) is one of the world’s most important commercial crops, expected to produce over 85% of the global sugar production over the coming decade and becoming increasingly important as a renewable bioenergy feedstock in emerging economies (OECD/FAO, 2025). However, sustainable sugarcane production faces persistent threats from biotic stresses, among which the African stalk borer, *Eldana saccharina* Walker (Lepidoptera: Pyralidae), is particularly destructive in the South African sugarcane industry. *E. saccharina* (hereafter referred to as eldana) causes extensive stalk damage through internal stalk boring, where larvae penetrate the sugarcane stalk and feed within the internodes. The concealed herbivory disrupts stalk tissue, reduces sucrose accumulation, leading to significant yield and quality losses (Rutherford, 2015). Although eldana does not directly cause red rot, the tunnelling damage it creates provides entry points for fungal pathogens, thereby predisposing the stalks to secondary infections and associated stalk rot (McFarlane et al., 2009).

To manage eldana populations, the South African sugarcane industry employs an area-wide IPM strategy that focuses on three primary components: regular field scouting, farming practices that reduce pest pressure, and bio-intensive methods that exploit ecological knowledge of pests and their natural enemies to enhance long-term sustainability. Farming practices aim to reduce plant stress and pest susceptibility through improved agronomic management, such as balancing soil nutrients (Keeping et al., 2014). Biological approaches include the deployment of resistant varieties and beneficial organisms that contribute to natural pest suppression. In addition, innovative strategies such as the “push-pull” system, which involves the use of repellent and attractive plants (Cockburn et al., 2014; Conlong et al., 2016; Mulcahy et al., 2023) and sterile insect technique (SIT) designed to suppress eldana populations to non-damaging levels, are being actively explored (Malinga, 2024; Walton and Conlong, 2016).

The South African Sugarcane Research Institute (SASRI) plant breeding program is responsible for releasing high-yielding, pest- and disease-tolerant sugarcane cultivars to the South African industry (Zhou, 2015). Resistance to eldana has been shown to be heritable, with family selection emerging as an effective breeding strategy (Zhou, 2016). Zhou (2016) found that the Gingindlovu research station in KwaZulu-Natal, South Africa, an area with consistently high eldana infestation pressure, produced parent genotypes whose families exhibited fewer bored stalks. Selection pressure at this site is further intensified using a long-cycle (18-month) selection program designed to identify cultivars capable of withstanding elevated pest pressure.

Despite considerable progress in the development of agronomic and biological control strategies, the molecular mechanisms underlying sugarcane resistance to eldana remain poorly understood. Little is known about the transcriptional defence responses activated in sugarcane following eldana herbivory, or how these responses differ between resistant and susceptible genotypes. Understanding the genes and regulatory pathways involved in these defence responses is essential for identifying molecular markers and candidate resistance genes that can accelerate breeding for insect-resistant cultivars.

In this study we used RNA sequencing (RNA-seq) to investigate the transcriptional responses of resistant and susceptible sugarcane genotypes following infestation by eldana. By comparing gene expression profiles between contrasting genotypes, we aimed to identify key defence related genes and pathways associated with resistance to eldana herbivory. We hypothesised that resistant genotypes exhibit a distinct transcriptional response characterized by the activation of defence signalling pathways, secondary metabolite biosynthesis, and structural defence mechanisms that limit larval feeding and tissue damage. The identification and characterization of genes induced during the defence response to eldana will provide new insight into molecular basis of insect resistance in sugarcane and can support the development of improved molecular breeding strategies for sustainable pest management.

## Materials and methods

### Eldana challenge RNA-seq study

A controlled pot trial was conducted at the South African Sugarcane Research Institute (SASRI) to compare the responses of two commercial sugarcane cultivars, N11 (susceptible) and N33 (resistant) to infestation by eldana. These cultivars were chosen based on susceptibility/resistance levels determined using resistance screening trial data (Keeping, 2006). Single-budded setts were grown in pots containing river sand under shade house conditions for nine months with optimal irrigation and fertilization. The experiment followed a two-factor randomised complete block design (cultivar × infestation status) with samples collected at two time points and three replicates per treatment – time combination giving 24 samples in total. Third-instar eldana larvae, reared on an artificial diet (Woods et al., 2020), were inserted into Eppendorf tubes attached to the ninth bud of two stalks per pot, while control plants received empty tubes One stalk was destructively sampled at 24 h (D1) after larval insertion and the second stalk was destructively sampled at 72 h (D2). A 20 mm stem tissue core surrounding the feeding site was excised, larvae were removed, and samples were snap-frozen in liquid nitrogen, ground to fine powder, and stored at –80 °C until RNA extraction.

### RNA extraction, library preparation and sequencing

Total RNA was extracted from stem tissue using the NucleoSpin RNA kit (Macherey-Nagel), followed by DNAse I treatment to remove residual genomic DNA. RNA was purified by precipitation and resuspended in DEPC-treated water. RNA quantity and purity were assessed using a NanoDrop ND-1000 spectrophotometer (NanoDrop Technologies, Wilmington, DE, USA), and RNA integrity was verified on agarose gels. Library preparation and sequencing was performed at the Agricultural Research Council, Onderstepoort, South Africa. Libraries were prepared from 1000 ng RNA using the TruSeq Stranded mRNA workflow, in which polyadenylated mRNA is enriched by oligo-dT capture. All samples were individually barcoded and sequenced as 125-bp stranded paired-end reads on an Illumina HiSeq 2500 platform.

### Sequence processing, transcriptome assembly and differential expression analysis

Raw sequencing reads were initially assessed using FastQC v0.11.5 (Andrews, 2010). Reads were then processed through a sequential filtering workflow. rRNA-derived reads were removed as a first step, followed by removal of plastome-derived reads using Mira v4.9.3 (Chevreux et al., 1999, 2004). Nuclear ribosomal reference sequences were obtained from GenBank accessions AH001756.2, AH001757.2 and AB285327.1. The plastome-derived reference sequences were based on the SP80–320 chloroplast genome (AE009947.2) and the sugarcane mitochondrial genome assembly described by Lloyd Evans et al. (2019). Adapter trimming was then performed with Trim Galore (Martin, 2011) in paired-end mode, retaining unpaired reads and applying a minimum read length threshold of 80 bp. The remaining reads were then quality filtered using Trimmomatic (Bolger et al., 2014) with Phred-33 encoding. Low-quality bases were removed from read ends, the first five bases were cropped, reads were trimmed using a sliding window approach and reads with average quality scores below 20 or lengths shorter than 36 bp were discarded. Cleaned reads were retained for downstream analyses. High-quality reads were assembled *de novo* using Trinity v2.4.0 (Grabherr et al., 2011). This approach was selected because the assembly was performed prior to the release of a sugarcane reference genome suitable for transcriptome-guided assembly. *De novo* assembly also reduces reference bias and allows the recovery of expressed cultivar and treatment specific transcripts. This is an important consideration given the highly polyploid and complex nature of the sugarcane genome. Assembly quality was subsequently refined using TransRate v1.0.3 (Smith-Unna et al., 2016), which removed poorly supported contigs under a TransRate optimised assembly score based on read-evidence metrics.

The high quality reads from all 24 libraries were aligned to the transcriptome using Bowtie2 v2.2.9 (Langmead and Salzberg, 2012). Transcript abundances were estimated with RSEM v1.3.0 (Li and Dewey, 2011) and expression matrices were generated using Trinity utility scripts. Differential expression analysis was performed using the Bioconductor package edgeR (Robinson et al., 2010) with trimmed mean of M-value (TMM) normalisation. Genes or isoforms exhibiting greater than a fourfold (2^2^) change in the expression and a false discovery rate (FDR) < 0.001 were considered significantly differentially expressed. Insect derived transcripts were identified based on their best BLASTX hit to a lepidopteran species against the ‘nr’ protein database (E-value cut-off of 1 x 10^-3^) and removed from further analysis using BLAST2GO (Conesa et al., 2005).

### Functional annotation and expression profiling

The assembled *Saccharum* hybrid eldana defence transcriptome was functionally annotated using EnTAP v0.10.4 (Hart et al., 2018). Coding regions were predicted using GeneMarkS-T v5.1 (Tang et al., 2015), and similarity searches were conducted against major protein databases using Diamond v0.9.9 (Buchfink et al., 2015), with an e-value cutoff of 1x10^–5^ and minimum alignment coverage threshold of 50%. This threshold reduces the retention of matches driven only by short conserved domains, while maintaining sufficient sensitivity for fragmented transcriptome assemblies. EnTAP was configured with taxon=Poaceae to favour taxonomically relevant hits from grasses during annotation. Sequences aligning to viruses, bacteria, arthropods, fungi, and vertebrates were removed as contaminants. Orthology assignment and Gene Ontology (GO) annotation were generated using InterProScan v5.45–80 (Jones et al., 2014) and EggNOG (Huerta-Cepas et al., 2016).

Hierarchical clustering was performed on the TMM-normalised expression matrix using the Trinity script define_clusters_by_cutting_tree.pl. This approach clusters transcripts according to similarity in their normalised expression profiles across samples, generating a dendrogram in which branch lengths reflect relative dissimilarity between expression patterns. Transcript dissimilarity is calculated using Euclidean distance and hierarchical clustering is performed using complete linkage. The resulting dendrogram was partitioned at 60% of the total tree height (--Ptree 60), resulting in eleven major clusters of co-expressed transcripts. Because the largest cluster (cluster 1) contained many transcripts and likely represented related but distinct expression trends, it was further subdivided by cutting the dendrogram at 50% of its maximum height (--Ptree 50) to resolve finer-scale expression patterns within the broader cluster.

Differentially expressed isoforms were further annotated using BLAST2GO v4.1.9 (Conesa et al., 2005) and NCBI’s *blastx* (Altschul et al., 1997) against the non-redundant (NR) database. Gene Ontology (GO) terms were assigned across the three major GO domains: molecular function (MF), cellular component (CC), and biological process (BP). Enriched GO terms among the differentially expressed transcripts were identified using Fisher’s exact test (p ≤ 0.01), and the GO annotations from the *Saccharum* hybrid eldana defence transcriptome.

### qPCR validation following a secondary eldana-challenge experiment

To validate the RNA-seq results and generate plant material more rapidly, a marcotting based stalk system was implemented. This approach provided a controlled and time-efficient means of maintaining mature, physiologically active stalk tissue for subsequent eldana challenge assays, mimicking the original experiment. Mature, eldana-free stalks from both cultivars were placed into metal canisters containing potting mix, with the basal stem ends submerged in a modified marcotting solution (James, 1980) composed of H_3_PO_4_ (0.8 mM), H_2_SO_4_ (0.49 mM), and HNO_3_ (0.35 mM), and supplemented with 2.64 g K_2_S_2_O_5_ per 10 L of acid solution. Marcotting was maintained for two weeks followed by a three-week recovery period under irrigation. The eldana challenge was conducted as described for the pot trial using the same susceptible and resistant cultivars included in the original RNA-seq experiment. Larval introductions were staggered so that all stalks were harvested at a single sampling event, while still representing the two challenge durations (24 h and 72 h after larval challenge). Four biological replicates were included for each time point. Unchallenged control stalks were sampled at the same harvest event and represented the corresponding 24 h and 72 h control treatment. For each challenged stalk, a 20 mm tissue core surrounding the larval entry site was harvested and immediately frozen for RNA extraction. For unchallenged controls, a corresponding 20 mm tissue core was collected from the equivalent stalk position.

Total RNA was extracted using Trizol (Invitrogen), treated with DNase I (Thermofisher), and purified using the RNeasy MinElute kit (Qiagen). First-strand cDNA synthesis was performed from 2 µg RNA, M-MLV reverse transcriptase (Promega) and oligo(dT) primers (Invitrogen). Three genes identified as differentially expressed in the RNA-seq dataset were selected for validation: a dir-like gene (DIR; TRINITY_DN163794_c0_g1) that was upregulated exclusively in the resistant cultivar, hydroxymandelonitrile lyase (HNL; TRINITY_DN229040_c0_g2), which showed strong upregulation in both cultivars and across both time points and β-cyanoalanine synthase (CAS; TRINITY_DN197535_c0_g1), which was upregulated ~2^3^ – 2^4^-fold in the early herbivory responses in both cultivars. Reference genes were evaluated using BestKeeper (Pfaffl et al., 2004) and NormFinder (Andersen et al., 2004), which identified actin (ACT), tubulin alpha (TUBA), and glyceraldehyde-3-phosphate dehydrogenase (GAPDH) as the most stable reference genes. The ACT primers were obtained from Ma et al. (2007), whereas the primers for all other genes were designed in-house. qRT-PCR was performed using PowerUp SYBR Green on a QuantStudio 5 system, with validated primer efficiencies (**Table 1**). Relative expression was calculated using ddCT method (Livak and Schmittgen, 2001) after normalization to the three stable reference genes. For samples in which no target amplification was detected by the final amplification cycle, the target was considered below the detection limit of the assay. To enable ddCT-based relative expression analysis, these non-detects were assigned a Ct value of 40, corresponding to the maximum cycle number.

**Table 1:** Primer sequences, concentrations, and qPCR performance parameters for the qRT-PCR assay conditions of target genes and reference genes.

Target	Final primer conc.	Oligonucleotide forward primer (5’-3’)	Oligonucleotide reverse primer (5’-3’)	Amplification efficiency (%)	R^2^	TM
CAS	150nM	TCCTTCAGCGTCAAAGACAGACC	GCCAAACCAATGCCCATGTT	108.5	0.997	81.4
HNL	300nM	CATCGACGGCTACAGCATCT	GTAGAAGGCGGTGCAAGGAT	101.3	0.999	88.6
DIR	300nM	GGATGATAGGTTTAAGGGATCCA	TGCACGGATATCAAGCTCCA	95.5	0.996	81.1
ACT	300nM	GACTCTGGTGATGGTGTCAGC	GGCTGGAAGAGGACCTCAGG	100.7	0.994	83.6
TUBA	200nM	AGGCCCAACTACTCCAACCT	TGAGGAGAGCATGAAGTGGA	108.4	0.990	82.7
GAPDH	300nM	GCCGTCAACGACCCCTTC	GGGTTCCTGATGCCAAAGACG	109.0	0.998	82.5

### Detection of a dirigent-like gene embedded in a presence/absence genomic locus

The dirigent-like gene (TRINITY_DN163794_c0_g1) induced exclusively in the resistant cultivar following eldana challenge, was extended to full length using an in-house bait-and-build pipeline, similar to that described in Evans and Joshi (2016). The Trinity transcript sequence was used as the initial bait to identify RNA-seq reads from the original Illumina dataset using Mira v4.9.3 (Chevreux et al., 2004). Recruited reads were then assembled using SPADES v3.5.0 (Bankevich et al., 2012) with multiple k-mer sizes and the transcript supplied as an untrusted contig, generating an extended transcript contig. The extended contig was used iteratively as a new bait sequence to recruit additional overlapping reads and extend the transcript further. The reconstructed sequence was further assessed for an intact coding region by open reading frame prediction. The amino acid sequence of the extended transcript was aligned against the sugarcane DIR protein sequences reported by Nobile et al. (2017), retrieved from the supplementary FASTA dataset associated with that study. The transcript matched ShDIR31 (scf7180000331296_g7159) with 100% amino acid identity over 99% query coverage, supporting its annotation as ShDIR31.

To determine whether the absence of ShDIR31 transcript detection in the susceptible cultivar reflected lack of expression or absence of the gene itself, genomic DNA was examined. The reconstructed transcript was validated at the genomic level by identifying the corresponding gene region in R570 reference genome (Garsmeur et al., 2018). Multiple primer pairs designed across approximately 13 kb region spanning and flanking the ShDIR31 locus (data not shown) confirmed consistent amplification in the resistant cultivar but failed to amplify in the susceptible cultivar, while control loci amplified successfully. Screening of an additional eight commercially grown sugarcane cultivars showed reproducible presence/absence patterns across multiple primer sets, supporting the interpretation that ShDIR31 occurs within a presence/absence variation (PAV) region.

### Molecular screening assay for the PAV region

A molecular screening assay was developed to assess the presence of PAV region in sugarcane cultivars. Genomic DNA was extracted from 100 mg leaf tissue using a Precellys bead-based homogeniser (Bertin Technologies) followed by purification with GeneJet plant genomic DNA purification kit (Thermo Fisher Scientific). Two genes within an 8 kb region were amplified using gene-specific primers *ShDIR31* (123bp) [5’-GGGACAGGAGAGTTTACCATG-3’/5’–CTTTGGTTCTTTGATAGGCACG-3’] and PAV gene 2 (910bp) [5’-TGACATGTGGGAGCCTGAAT-3’/5’-GAAGTGGCATCCTTGTCGTC-3’]. A reference gene was co-amplified using the primers RNApol (442bp) [5’-TCCTGTTGATGGGAACTGCT-3’/5’-ATAGTCCCTTCCAAGAGCGG-3’] to serve as a positive DNA control. PCR reactions (20 µl) contained 50 ng genomic DNA, 0.4 µM of each *ShDIR31* primer, 0.4 µM of each PAV gene 2 primer, 0.2 µM of each RNApol primer, 1x PCR buffer, 0.4 mM dNTPs and 0.1 µl GOTaq DNA polymerase (Promega, Madison, WI, USA), with nuclease-free water added to volume.

Thermocycling was carried out with an initial denaturation at 95 °C for 3 min; 30 cycles of 95 °C for 30 s, 60 °C for 30 s, and 72 °C for 1 min; followed by a final extension at 72 °C for 2 min. PCR products were separated by agarose gel electrophoresis to determine the presence or absence of the PAV region.

The screening assay was validated across a panel of fifty-eight South African commercially released sugarcane cultivars. Cultivars which contained the PAV region were considered positive and were scored as MS: 1, whilst those without the PAV region (no amplification) were scored as MS: 0, despite successful amplification of the reference loci. The results were consistent when the same cultivars were collected from different locations.

### Assessment of eldana resistance using plant breeding data and variety evaluation pot-trials

Prior to commercial release, sugarcane cultivars are evaluated for eldana resistance in multilocation variety trials along with controlled pot-trials following the standard procedure described by Joshi et al. (2012) and Keeping (2006), respectively. All genotypes included in a controlled pot trial (n=74) aimed at assessing eldana resistance in final stage breeding trials were assessed for marker score (MS: 0/1) The pot trial was established according to Keeping (2006) and the damage metric, percent internodes damaged (PID) for all replicated genotypes, was recorded.

The data were analysed in the Statistical Analysis System (SAS version 9.4) using a linear mixed model. Genotype was fitted as a random effect, and best linear unbiased predictions (BLUPs) were extracted. PID BLUP values were standardised to generate PID_z values by subtracting the trial mean from each genotype PID BLUP and dividing by the trial standard deviation. Negative PID_z values indicate below average internode damage and therefore greater resistance, while positive PID_z values indicate higher than average internode damage and greater susceptibility. Genotypes with a PID_z damage values ≤ −0.3 were classified as resistant (R), genotypes with values > −0.3 and < +0.5 were classified as intermediate (I), and genotypes with values ≥ +0.5 were classified as susceptible (S).

The association between *ShDIR31* marker score (MS: 0/1) and resistance classification was assessed using the SAS software package. Resistance classification was analysed as a three-level categorical variable (R/I/S). Contingency tables were generated and the association between marker score and resistance classification was evaluated using the Pearson chi-square test.

To support the categorical analysis, the continuous damage index was also compared between MS: 1 and MS: 0 genotypes. Welch’s t-test was used to compare mean damage values between marker score groups, and the Mann-Whitney test was used as a non-parametric comparison of the damage distributions. These analyses were used to determine whether MS: 1 genotypes showed a shift towards reduced eldana damage relative to MS: 0 genotypes.

## Results

Infestation success in the eldana challenge pot trial was confirmed prior to RNA extraction for the RNA-seq study. All challenged plants were confirmed to contain an eldana larva at harvest. Where larvae were located outside the stem, successful feeding was supported by visible damage, including bored or chewed buds or the presence of frass. Where larvae were found within the stalk tissue, they were removed before sample processing.

### RNA-seq data generation and *de novo* transcriptome assembly

A total of 340,783,248 paired-end raw reads (125 bp) were generated from the 24 sequencing libraries. Raw sequence reads were deposited in the NCBI Sequence Read Archive under BioProject accession PRJNA760978. Ribosomal and plastome derived reads were removed and the remaining reads were trimmed to eliminate sequencing adapters and low-quality regions. After pre-processing, the dataset comprised of 279,132,362 high quality paired-end reads. The number of paired reads per sample ranged from 5.2 million to 23.8 million, with an average of 11.6 million reads per sample. This variation in sequencing depth was accounted for during downstream analysis by library-size normalisation in edgeR. Lowly expressed transcripts were filtered using a minimum CPM threshold across biological replicates before differential expression testing. These steps reduce the influence of unequal library sizes and ensure that differential expression estimates are based on normalised expression rather than raw read counts. A preliminary *de novo* transcriptome assembly, differential expression analysis and functional annotation of differentially expressed (DE) isoforms enabled the identification of transcripts originating from eldana larva contamination. These contaminating transcripts were subsequently used to bait and remove corresponding reads from the original libraries, yielding a refined dataset of 267,672,216 paired-end reads.

A *de novo* eldana-challenged sugarcane transcriptome was assembled generating 863,130 contigs with an average length of 541 bp, an N50 of 763 bp and an overall raw-read alignment rate of 96.29%. The high number of contigs is likely to reflect alternative isoforms, fragmented assemblies and homoeologous sequence variants, which are expected in a highly polyploid crop such as sugarcane. Assembly quality was further improved using TransRate. The resulting TransRate score remained modest but improved from 0.135 to 0.224 after filtering, indicating an improvement in read-supported assembly quality. The relatively low score likely reflects the complexity of the *de novo* sugarcane transcriptome assembly, including the redundancy inherent to complex genomes. The filtered assembly contained 610,733 higher confidence contigs with an improved N50 of 875 bp, which was retained for downstream analyses. BUSCO analysis using the embryophyte_odb12 dataset identified 81% of single copy BUSCOs (from a total of 2026), with only 253 BUSCOs (18.9%) missing. The EnTAP annotation of the *de novo* assembled transcriptome (**Supplementary Material S1**) predicted 146,789 putative coding sequences, including 56,777 complete genes, indicating a substantial representation of full-length transcripts. These predicted coding sequences represent transcripts with identifiable open reading frames, whereas functional annotation was assigned based on similarity searches or gene family classification. Similarity searches across multiple protein databases resulted in 93,757 transcripts with significant alignments. The majority of top hits were to Poaceae species, particularly *Sorghum bicolor, Zea mays* and *Panicum hallii*. Contaminant screening identified 14.3% of sequences as potential non-plant contaminants, the majority from arthropoda and bacteria. Gene family assignments were made for 116,151 sequences, while 116,140 sequences received Gene Ontology (GO) terms and approximately 27,000 were linked to KEGG pathways. Overall, 118,399 transcripts were successfully annotated through similarity and/or gene family assignment, providing a robust foundation for downstream functional and comparative analyses.

### Differential gene expression in response to eldana herbivory

**Figure 1:**
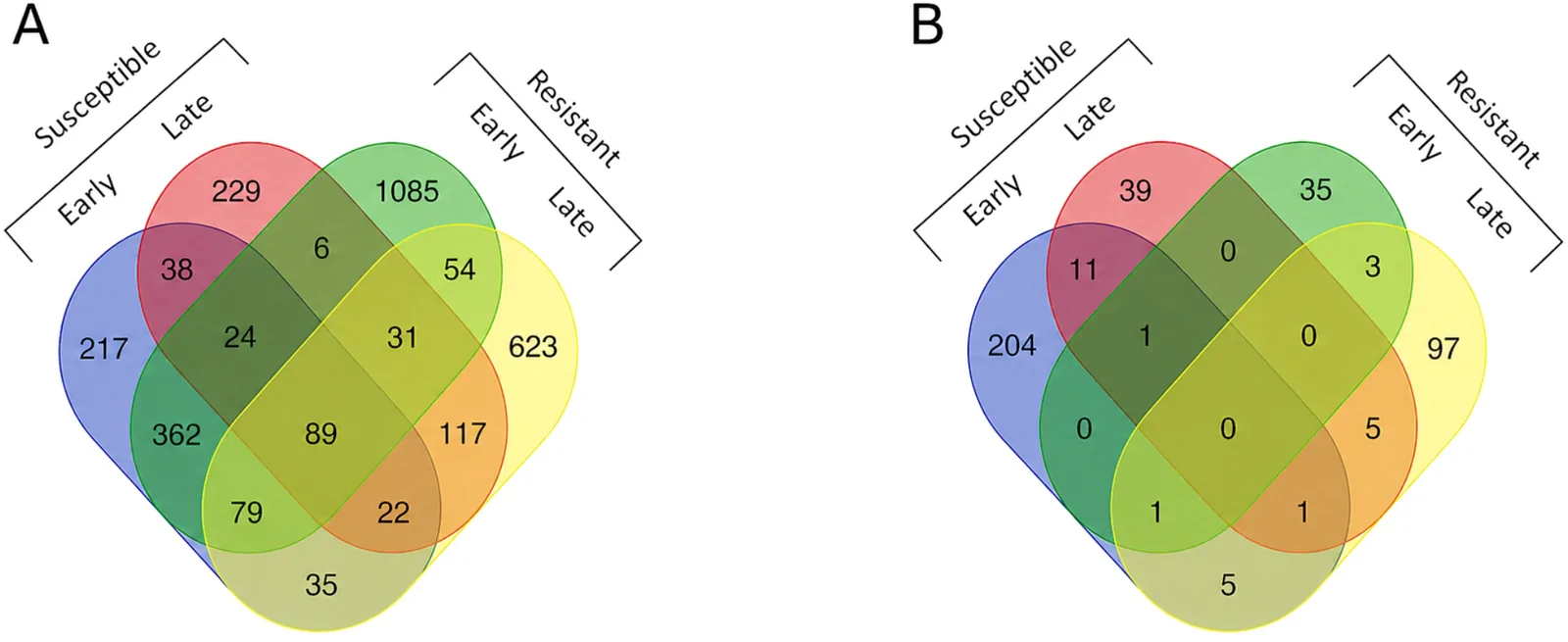
Number of differentially expressed genes that were **(A)** upregulated and **(B)** downregulated in the early and late response to eldana herbivory in the resistant and susceptible sugarcane cultivars. Venn diagrams were generated using the Venn diagram tool (http://bioinformatics.psb.ugent.be/webtools/Venn/).

A total of 4,634 differentially expressed genes (DEGs) were identified by pairwise-comparison of the eldana-challenged and control sugarcane plants from the two cultivars differing in resistance to the stem-boring pest. Overall, the resistant cultivar mounted a stronger transcriptional response than the susceptible cultivar (**Figure 1**). In the early resistant response, 1,770 DEGs were identified, of which 98% were upregulated and 2% were downregulated. In comparison, the early susceptible response comprised 1,089 DEGs with 80% upregulated and 20% downregulated.

Amongst the upregulated genes identified by pairwise comparison, more than twice as many were detected in the early resistant response (1,730 DEGs) as compared to the early susceptible response (866 DEGs). A similar trend was observed in the late responses, where 1,050 genes were upregulated in the resistant cultivar compared with 556 genes in the susceptible cultivar. In contrast, more genes were downregulated in the early susceptible response (223 DEGs) than in the early resistant response (40 DEGs). It should be noted that these DEG counts reflect only transcripts identified through pairwise differential expression analysis and include both annotated and unannotated sequences.

Overall, these patterns are consistent with a more rapid activation of a defence-associated response in the resistant cultivar, whereas the susceptible cultivar exhibits a comparatively weaker transcriptional response and a greater degree of early transcriptional repression (**Figure 1B**). While both cultivars shared a core set of differentially expressed genes, there was little overlap observed among downregulated genes, suggesting that they employ distinct regulatory strategies in response to herbivory.

### Co-expression clustering of differentially expressed genes

**Figure 2:**
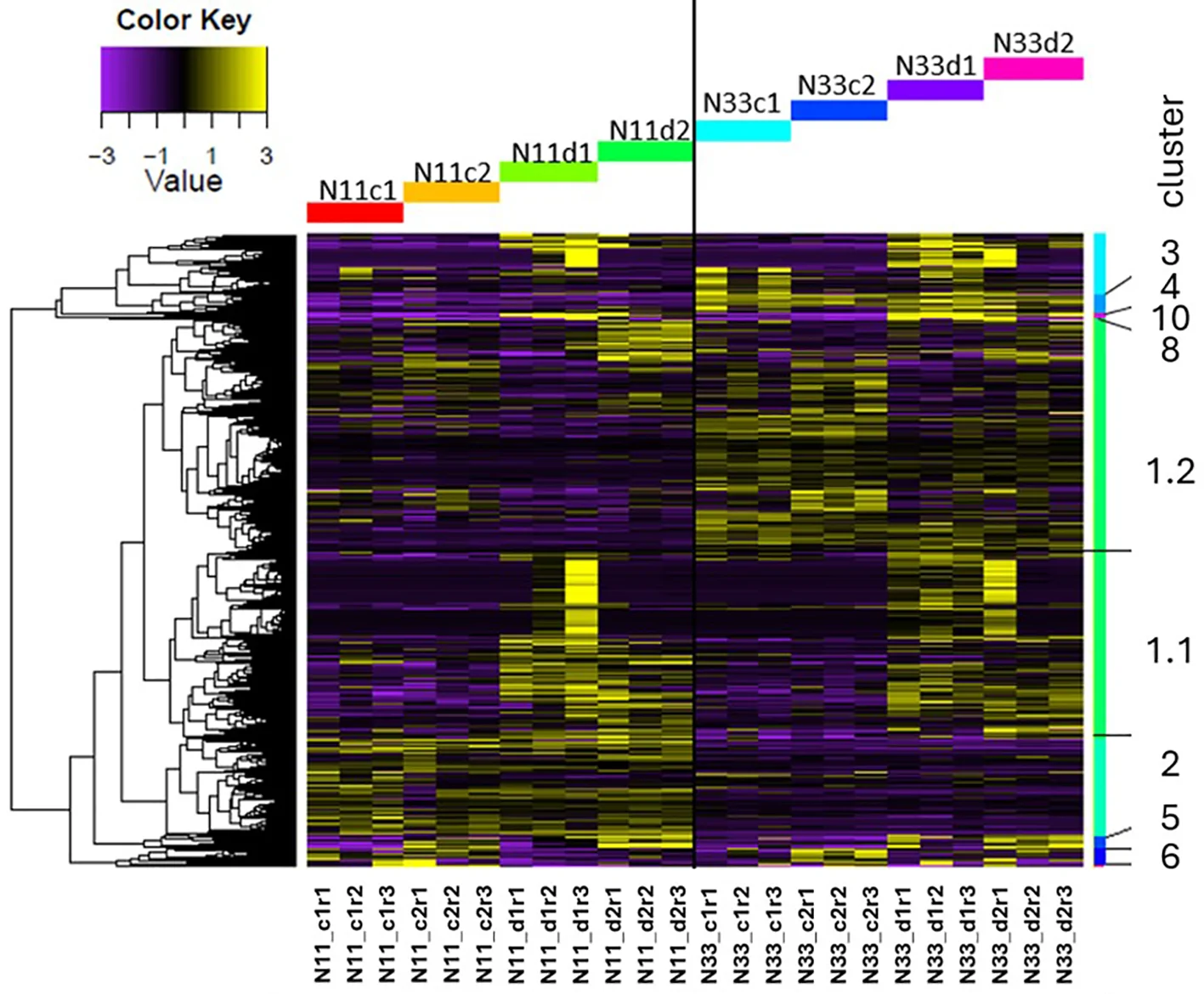
Hierarchical clustering analysis of differentially expressed genes. Rows represent individual genes. Upregulated expression is displayed in yellow and downregulated expression in purple. The coloured vertical bar and labels on the right indicate the major clusters that were cut from the dendrogram. N11: susceptible cultivar; N33: resistant cultivar; c1: untreated control early timepoint; c2: untreated control late timepoint; d1: early challenged timepoint; d2: late challenged timepoint; r: replicates.

Hierarchical clustering groups genes according to shared expression patterns across genotypes, treatments and time points (**Figure 2**). In the heatmap, each row represents a gene and each column, a sample. Higher expression values are shown in yellow, and lower expression levels in purple, with the colour scale constrained from -3 to 3 to enhance the visibility of strongly regulated genes. Cutting the dendrogram generated eleven co-expression clusters where genes with similar expression patterns, grouped together. Three clusters were excluded from further analysis because they contained very few genes or were identified as containing contaminant sequences. The largest cluster (cluster 1), containing 9,324 DEGs, was further subdivided into two groups (clusters 1.1 and 1.2) by applying a further cut at 50% of the tree’s maximum height. This separated genes that were predominantly induced during the early response (cluster 1.1: 4,162 genes) from those induced mainly during the late response (cluster 1.2: 5,162 genes).

**Figure 3:**
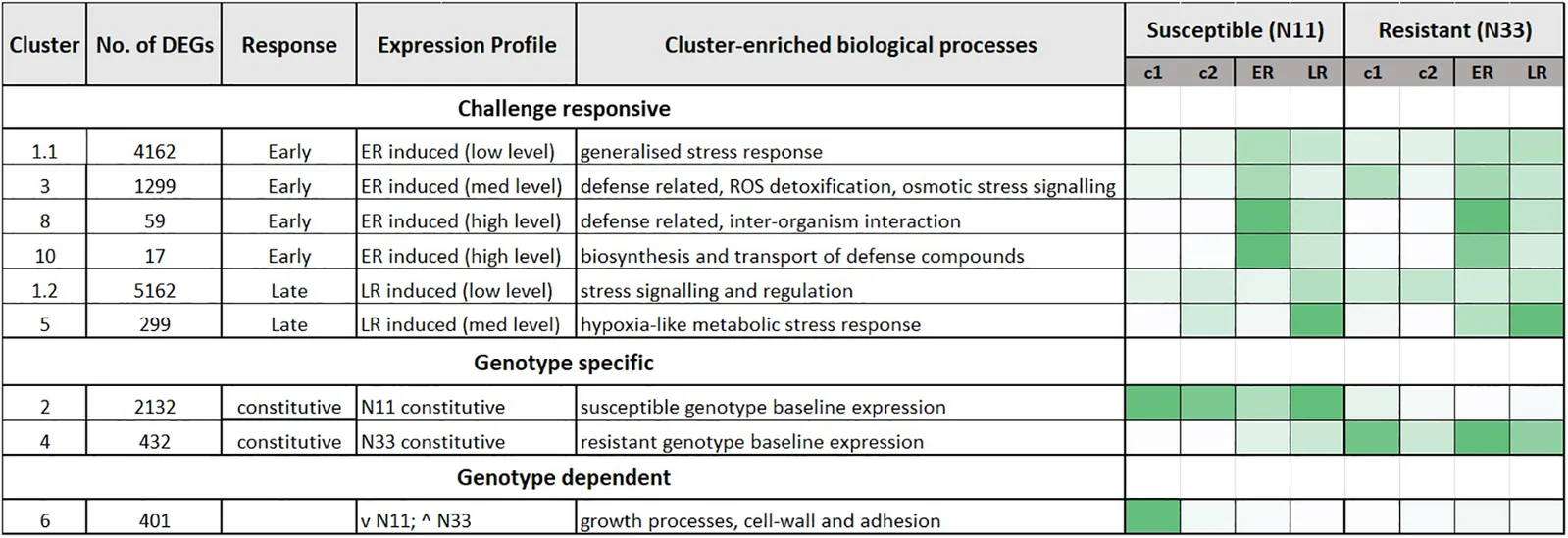
Summary of gene expression clusters identified following eldana challenge. Differentially expressed genes were grouped into expression clusters. Green shading indicates the relative contribution of each sample group to the total expression within each cluster, with darker shading representing a higher contribution. Sample groups are shown separately for the susceptible cultivar N11 and resistant cultivar N33. c1: untreated control early timepoint, c2: untreated control late timepoint, ER: early response (early challenged timepoint), LR: late response (late challenged timepoint).

For each expression cluster, normalised expression values were averaged across biological replicates within each cultivar, treatment and time-point combination. These group means were then expressed relative to the total expression for that cluster and visualised as a heatmap (**Figure 3**). Analysis of the cluster mean expression profiles across genotypes and conditions revealed broader transcriptional patterns. For interpretative clarity, the clusters were grouped into three higher order response classes: challenge-responsive modules, genotype-specific modules, and genotype-dependent divergent modules. Gene Ontology (GO) enrichment analysis of co-expressed annotated DEGs in each cluster revealed distinct functional response classes.

#### Early challenge responsive modules

Clusters (1.1, 3, 8, and 10) were induced in both genotypes following herbivory, although they differed in response magnitude and functional emphasis. Cluster 1.1 (4,162 genes) and cluster 3 (1,299 genes) were enriched for general stress related processes, including reactive oxygen species detoxification, defence metabolite production, stress hormone signalling and cell wall organisation. Cluster 3 was additionally enriched for osmotic and water stress responses, likely reflecting physiological stress associated with larval feeding. Clusters 8 and 10 (59 and 17 genes, respectively) showed strong induction in both cultivars at both time points. Cluster 8 was enriched for defence-related processes involved in inter-organism interactions and was dominated by degradative and detoxification enzymes whilst cluster 10 was strongly enriched for secondary metabolite biosynthesis, including volatile compounds, terpene metabolism, and cyanogenic glycoside pathways.

#### Late challenge responsive modules

Clusters 1.2 and 5 were predominantly associated with the late response. Cluster 1.2 (5,162 genes) encompassed both up- and downregulated genes and was enriched for stress signalling, regulatory functions, carbohydrate metabolism, and secondary metabolite biosynthesis and transport, with ‘defence response’ and ‘response to stress’ as dominant GO terms. Cluster 5 (299 genes) was enriched for hypoxia-related processes and contained genes induced in the resistant early resistant response and in both genotypes at the late time point, likely reflecting localised tissue damage and reduced gas exchange following larval feeding.

#### Genotype specific and genotype dependent modules

Clusters 2 and 4 reflected constitutive transcriptional differences between cultivars. Cluster 2 (2,132 genes) showed higher basal expression in the susceptible cultivar and was enriched for defence response, DNA metabolism, recombination, and transposon-associated processes, suggesting immune activation alongside stress-related genome maintenance. Cluster 4 (432 genes) comprised genes constitutively expressed in the resistant cultivar but absent or weakly expressed in the susceptible cultivar. This gene set was enriched for genome maintenance, DNA modification, and transposon-associated functions. Cluster 6 (401 genes) displayed higher basal levels of expression in the resistant cultivar and sustained downregulation in the susceptible responses. This cluster was enriched for cell wall and adhesion-related processes, indicating repression of growth-associated functions during herbivory and a shift towards defence, stress metabolism, and rapid signalling.

### Phytohormone and herbivore-induced defence responses

Analysis of DEGs revealed strong induction of pathogenesis-related (PR) defence genes associated with salicylic acid (SA), jasmonic acid (JA) and ethylene (ET) signalling pathways following eldana herbivory (**Supplementary Material S2**).

Herbivory triggered strong induction of SA-associated pathogenesis-related (PR) genes, particularly in the early susceptible response. Members of the PR-1, PR-2, and PR-5 families were prominently upregulated. PR-1 transcripts showed strong early induction in the susceptible cultivar, consistent with a transcriptional signature associated with SA-dependent signalling. Multiple PR-2 β-1,3-glucanases and PR-5 thaumatin-like proteins were also upregulated early, with some PR-5 genes showing sustained expression into the late response. These SA-responsive genes were not detected as differentially expressed in the late resistant response under the applied thresholds.

The JA-responsive genes included members of the PR-3, PR-4 and PR-6 families. Genes encoding chitinases (PR-3) were prominently upregulated in both cultivars, particularly during the early responses. Protease inhibitor genes (PR-6), including α-amylase/trypsin inhibitors, Bowman–Birk–type inhibitors and maize proteinase inhibitors (MPI), were also strongly induced across both genotypes and displayed persistent expression into the late response. A large suite of peroxidase genes (PR-9) functioning at the interface between SA- and JA-mediated defence pathways, was also differentially expressed. While some peroxidases showed strong induction in the susceptible response, the resistant cultivar more frequently exhibited sustained expression across time points or late-stage induction, suggesting a sustained transcriptional response involving oxidative defence activity and cell-wall associated peroxidase activity following larval feeding.

In addition to PR-mediated defences, herbivory induced genes involved in cyanogenic metabolism and secondary metabolite modification. Multiple CYP79A1 isoforms, encoding tyrosine *N*-monooxygenases, and catalysing the first committed step in cyanogenic glucoside biosynthesis, were strongly induced in both cultivars across early and late responses. Downstream components of the cyanogenic pathway were also differentially expressed, including hydroxymandelonitrile lyase (HNL), which releases hydrogen cyanide upon tissue damage. HNL transcripts showed a similar pattern with strong induction in both genotypes with expression generally maintained across time points. In parallel, a gene encoding β-cyanoalanine synthase (β-CAS), which detoxifies released cyanide, was induced in both cultivars but showed lower magnitude than CYP79A1 and HNL, consistent with coordinated transcriptional induction of genes associated with both the bioactivation and detoxification arms of cyanogenic defence.

Several WRKY transcription factors were also induced, including WRKY24, WRKY53, WRKY71, WRKY72, and WRKY75. Of these, WRKY75 and WRKY53, were strongly upregulated in the late susceptible response, whereas, WRKY72 showed pronounced induction in the late resistant response, with low-level basal expression observed only in the resistant control plants.

Sesquiterpene biosynthesis was strongly activated following herbivory. Genes encoding (E)-β-caryophyllene synthase and α-humulene synthase genes were upregulated in both genotypes and at both response stages. Multiple β-sesquiphellandrene synthase–like genes, largely within cluster 8, were induced in both genotypes but more strongly in the susceptible cultivar. A sesquiterpene cyclase gene was also induced specifically in the early susceptible response. The upregulation of these genes suggests transcriptional activation of sesquiterpene biosynthetic capacity after larval feeding. Two genes encoding premnaspirodiene oxygenase (CYP71) were strongly induced in the susceptible cultivar but showed little or no expression in the resistant genotype, suggesting genotype-specific transcriptional regulation of enzymes putatively involved in sesquiterpene modification.

Collectively, these data indicate that eldana herbivory activates a strong volatile sesquiterpene biosynthetic response in sugarcane, dominated by caryophyllene-, humulene-, and sesquiphellandrene-related pathways. The higher magnitude of induction observed in the susceptible cultivar may indicate stronger transcriptional investment in volatile-associated pathways, whereas the resistant cultivar displays more moderate or selective activation of these pathways, consistent with a transcriptional profile in which structural and direct chemical defence-related genes are more prominent.

In *Zea mays*, SA biosynthesis has been shown to depend on phenylalanine ammonium lyase (PAL), which produces the SA precursor benzoic acid from the early phenylpropanoid biosynthetic pathway (Yuan et al., 2019). In the present study, PAL upregulation in the susceptible cultivar supports the possibility of a stronger SA-associated transcriptional response.

Multiple linoleate 9S-lipoxygenases (9-LOX) genes were differentially expressed in both cultivars. 9-LOX1 and 9-LOX2 were differentially expressed in the early and late resistant response to herbivory, whilst they were mostly detected only in the late susceptible response. Differential regulation of TIFY/JAZ domain proteins indicated turnover of JA repressors in both cultivars.

Several genes involved in ethylene biosynthetic and signalling pathways were also differentially expressed in response to herbivory. A gene encoding 1-aminocyclopropane-1-carboxylate (ACC) synthase (ACS) was upregulated only in the late resistant response, whilst genes encoding ACC oxidase (ACO), which catalyses the final step of ethylene biosynthesis, were identified among the highly induced Cluster 8 genes and were present in both resistant and susceptible early responses to herbivory. Several ethylene response factors (ERFs), including ERF1, were differentially expressed in both resistant and susceptible defence responses.

### Cell wall biosynthesis and remodelling

Hierarchical clustering and Gene Ontology enrichment analyses revealed a strong over-representation of genes associated with cell wall organisation, secondary cell wall (SCW) biosynthesis, and cell wall remodelling among the DEGs, particularly in the resistant cultivar following eldana herbivory.

In the early resistant response, key transcriptional regulators of SCW formation were induced, including multiple members of the NAC and MYB transcription factor families that act as upstream regulators of SCW biosynthetic pathways. NAC master regulators were induced specifically in the early resistant response, with little evidence of induction in the susceptible cultivar. In contrast, MYB transcription factors were upregulated in the early response of both cultivars, but sustained upregulation was largely restricted to the resistant cultivar.

Consistent with activation of this regulatory network, genes encoding core components of cellulose biosynthesis were strongly induced, particularly at the late time point in both cultivars. These included multiple cellulose synthase (CESA) isoforms and sucrose synthase gene involved in UDP-glucose supply, indicating increased carbon flux towards cellulose deposition during the defence response.

Extensive induction of the phenylpropanoid and monolignol biosynthesis pathways was also observed. Multiple isoforms of phenylalanine ammonia-lyase (PAL), cinnamate-4-hydroxylase (C4H), 4-coumarate-CoA ligase (4CL), hydroxycinnamoyl transferase (HCT), cinnamoyl-CoA reductase (CCR), caffeoyl-CoA O-methyltransferase (CCoAOMT), and caffeoyl shikimate esterase (CSE) were differentially expressed. Genes encoding oxidative polymerisation enzymes, including numerous peroxidases and laccases, were also upregulated, with several laccases showing stronger late induction in the resistant cultivar, consistent with enhanced lignification at sites of herbivore damage.

Genes involved in xylan biosynthesis and secondary wall matrix reinforcement were preferentially induced in the resistant cultivar. Members of the IRREGULAR XYLEM (IRX) family and glucuronoxylan-modifying enzymes (GUX) were upregulated predominantly in the resistant response, suggesting increased expression of genes associated with hemicellulose–cellulose–lignin matrix reinforcement in resistant stem tissue. In addition, cell wall remodelling genes displayed contrasting expression patterns between cultivars: xyloglucan endotransglycosylase/hydrolase (XTH8) genes were strongly induced early in the resistant response, whereas genes encoding β-expansins were transcriptionally repressed in the late resistant response.

Together, these expression patterns are consistent with extensive transcriptional reprogramming of cell wall biosynthesis, lignification, and remodelling pathways, with earlier activation of regulatory transcription factors and stronger induction of genes associated with reinforcement of SCW components in the resistant cultivar. The full list of genes, expression clusters, and genotype and time-specific fold changes supporting these conclusions is provided in **Supplementary Materials S2** and **S3**.

### Validation of RNA-seq data

**Figure 4:**
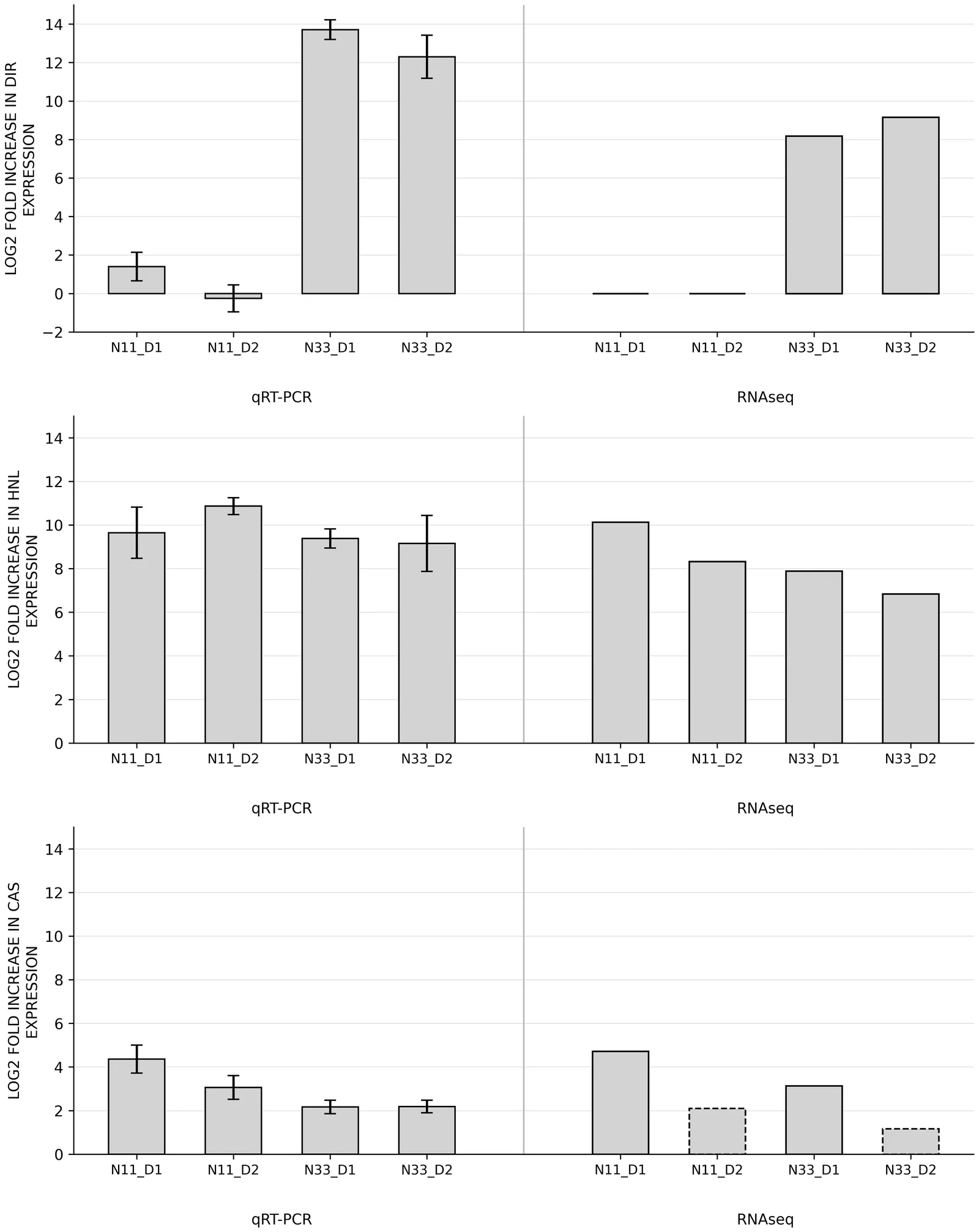
Comparison of qRT-PCR and RNA-seq estimates of gene induction following eldana challenge. The log2 fold increases in DIR, HNL and CAS gene expression were calculated for challenged samples relative to their corresponding unchallenged controls. qRT-PCR values were obtained from the marcotting-based challenge trial, while RNA-seq values were derived from the original pot-trial experiment. N11: susceptible cultivar; N33: resistant cultivar; D1: early challenged timepoint; D2: late challenged timepoint.

The expression of three DEGs identified in the RNA-seq study were validated with samples from a marcotting trial, challenged with eldana. Although this did not constitute a direct one to one comparison because the source of the plant material differed between experiments, the qRT-PCR results were highly concordant with the RNA-seq data (Pearson correlation analysis gave r = 0.944), confirming both the direction and magnitude of the expression changes observed in the RNA-seq dataset. qRT-PCR revealed strong induction of the target genes: HNL expression increased by more than 2^8^-fold, while β-CAS expression increased by approximately 2^2^ – 2^4^-fold (**Figure 4**). Although the β-CAS was not classified as differentially expressed in the late responses due to p-values exceeding the significance threshold, its original expression values were retrieved for comparison and are indicated in the graph with a dotted border. The qRT-PCR differential expression results also confirm that the dirigent-domain containing gene was strongly induced by herbivory in the resistant cultivar only. Because non-detects were assigned a Ct value of 40, fold-change estimates involving these samples should be interpreted cautiously. Emphasis should rather be placed on the consistent presence/absence of detectable amplification and the direction of expression differences, rather than on the exact fold-change values for comparisons involving non-detects in the DIR assay.

### A PAV region containing a dirigent-like gene and its relationship with eldana resistance

The dirigent-like gene that was induced by eldana herbivory and expressed only in the resistant cultivar was identified as *ShDIR31* (Nobile et al., 2017). The gene spans 768 bp and contains three exons and two introns, which are transcribed into a 531-nucleotide mRNA encoding a protein of 177 amino acids.

Resistance status was assessed on a standardized percent internodes damaged value (PID_z) for the trial (**Supplementary Material S4**). The presence of the ShDIR31 PAV region was significantly associated with resistance status (χ²(2) = 13.08, p = 0.0014). Among MS: 1 genotypes, 56.4% were classified as resistant, 30.8% as intermediate and 12.8% as susceptible. In contrast, among MS: 0 genotypes, 22.9% were classified as resistant, 28.6% intermediate and 48.6% susceptible. When only the clear resistant and susceptible classes were considered, 81.5% of the MS: 1 genotypes were resistant, and 68% of the MS: 0 genotypes, were susceptible This association was significant using both Pearson’s chi-square test (χ²(1) = 13.02, p = 0.0003) and Fisher’s exact test (two-sided p = 0.0006). The continuous PID values were significantly lower in MS: 1 genotypes using both Welch’s t-test (p = 0.0005) and the Mann-Whitney test (p = 0.0009), supporting an association between marker presence and reduced internode damage. Amongst genotypes clearly classified as either resistant or susceptible, MS: 1 genotypes had 9.35 times higher odds of being resistant than MS: 0 genotypes.

## Discussion

In this study, RNA-seq was used to investigate the transcriptional responses of two sugarcane cultivars differing in their resistance to *Eldana saccharina*. A controlled challenge experiment was conducted, in which eldana larvae were forced to bore into the sugarcane stems, and samples were collected at two timepoints to capture both early and late responses to the herbivory. To resolve the transcriptional landscape underlying sugarcane responses, DEGs were grouped into co-expression clusters based on shared expression patterns across treatments and genotypes. This approach enabled the identification of coordinated gene networks associated with both genotype-specific constitutive expression and herbivory-induced defence. Constitutively expressed genes are important as they reflect pre-existing differences in defence readiness and signal perception between resistant and susceptible genotypes. However, the primary focus of this analysis was on genes and pathways differentially expressed following eldana challenge, as these are most likely associated with active defence responses to herbivory.

### Hormonal and chemical defence networks activated by eldana herbivory

When attacked by herbivorous insects, plants activate defence hormone signalling pathways and deploy chemical defences that either deter feeding directly or promote the release of herbivore induced plant volatiles (HIPVs) that attract natural predators of the herbivore (Dicke et al., 2009). Because the production of defence proteins and secondary metabolites carries substantial metabolic costs, these responses are typically tightly regulated (Herms and Mattson, 1992). Consistent with this, our clustering analysis revealed discrete gene modules that were selectively activated in response to herbivory, highlighting inducible transcriptional responses associated with sugarcane defence.

Among the pathogenesis-related (PR) genes induced by herbivory, the rapid and strong activation of protease inhibitors (PR-6) is consistent with a jasmonic acid (JA) -associated anti-herbivore defence response. These inhibitors interfere with the insect midgut proteases, reducing nutrient acquisition and slowing larval development (Lawrence and Koundal, 2002; Zhao et al., 2019). Genes encoding multiple protease inhibitors were mainly identified in the strongly induced early defence clusters (clusters 3 and 8) and remained upregulated into the late response in both the resistant and susceptible cultivars.

Cyanogenic metabolism was also predominantly induced. Dhurrin, first identified in *Sorghum bicolor*, functions both as a nitrogen reserve and as a deterrent to herbivory (Tattersall et al., 2001; Krothapalli et al., 2013). Dhurrin biosynthesis is known to be induced by wounding and methyljasmonate treatment (Sohail et al., 2023). In this study, a gene encoding tyrosine N-monooxygenase (CYP79A1), which catalyses the first and rate-limiting step in the biosynthesis pathway, was strongly induced following herbivory. Upon tissue damage, β-glucosidases and hydroxymandelonitrile lyase (HNL) convert cyanogenic precursors into hydrogen cyanide, whilst β-cyanoalanine synthase (β-CAS) prevents self-toxicity by detoxifying the released cyanide. The coordinated induction of CYP79A1, HNL, and β-CAS in both cultivars suggests transcriptional activation of genes associated with both the bioactivation and detoxification branches of a cyanogenic defence response against eldana.

In addition to direct defences, genes involved in herbivore-induced plant volatile (HIPV) biosynthesis were strongly induced, particularly within cluster 10. Volatiles such as (E)-β-caryophyllene play a key role in indirect defence by recruiting natural enemies of herbivores (Rasmann et al., 2005; Köllner et al., 2008). The induction of terpene synthases and methyltransferases suggests that sugarcane may respond to eldana feeding by increasing expression of genes involved in volatile biosynthesis, which could contribute to complex volatile blends that may mediate interactions among plants, herbivores and predators. Genotype-specific differences in volatile biosynthetic pathways were also evident. The induction of premnaspirodiene oxygenase genes exclusively in the susceptible cultivar suggests differential deployment of phytoalexin-like pathways, which may reflect genotype-specific differences in secondary metabolite pathway regulation.

These induced defences are closely linked to jasmonic acid (JA) signalling. In the JA pathway 13-lipoxygenase (13-LOX) catalyses the first committed step by oxygenating α-linolenic acid at the carbon-13 position. The 9-LOX pathway represents a parallel branch of oxylipin metabolism that oxygenates fatty acids at the carbon-9 position. Whilst 13-LOXs are well established in JA biosynthesis, the function of 9-LOX-derived oxylipins are less clearly understood (Wasternack and Song, 2017). In *Arabidopsis thaliana*, 9-LOXs have been implicated in brassinosteroid signalling and cell-wall-mediated defence responses (Vellosillo et al., 2007; Marcos et al., 2015). More recent studies support an important role for 9-LOX derived oxylipins in herbivore defence, with mutants lacking these genes showing increased insect damage and improved herbivore performance (Yuan et al., 2023; Jimenez-Aleman and Jander, 2023). Consistent with this, multiple 9-LOX genes were induced by eldana herbivory in both cultivars. Notably, multiple 9-LOX1 and 9-LOX2 genes were predominantly activated in the resistant cultivar during both early and late responses, whereas, activation in the susceptible cultivar was delayed until the late stage. This is particularly interesting, because in rice, Os9-LOX1 has dual 9- and 13-LOX positional specificity and contributes to the herbivore-induced JA burst as well as the cross talk between JA and salicylic acid (SA) in resistance to both chewing and phloem feeding herbivores (Wang et al., 2008; Zhou et al., 2014). The upregulation of these genes in the response to eldana is consistent with involvement of JA-associated signalling.

A second noteworthy observation was the induction of 9-LOX3 in the susceptible response. In rice, loss of 9-LOX3 confers increased resistance to insect herbivory, with reduced damage attributed to the loss of induced susceptibility, rather than enhanced defence (Zhang et al., 2007; Tang, 2016). LOX3 has also been implicated as a susceptibility factor for several fungal pathogens, including *Fusarium verticilloides* (Battilani et al., 2018). Its induction only in the susceptible cultivar therefore suggests that this gene raises the possibility that it is associated with an oxylipin branch linked to susceptibility, although functional validation would be required.

Further evidence for JA signalling was provided by the differential expression of several TIFY/JAZ transcripts, particularly in the early resistant response. JAZ transcript abundance is commonly interpreted as an indirect molecular marker for increased JA signalling. JAZ proteins repress JA responses by inhibiting JA responsive transcription factors (TFs). But JA also triggers a negative feedback loop in which rising hormone levels promote increased expression of JAZ genes (Pauwels et al., 2010). Under elevated JA conditions, JAZ proteins are ubiquitinated and degraded by the proteasome, thereby releasing the transcriptional repression and allowing defence activation (Howe et al., 2018). The occurrence of the multiple JAZ genes among the early responsive DEGs is therefore consistent with activation of JA signalling during eldana herbivory.

Differential expression of ethylene (ET) biosynthetic and response genes suggests possible involvement of ET-associated signalling. JA and ET are known to act synergistically in defence against necrotrophic pathogens and chewing herbivores, and ET plays an important role in fine tuning defence responses through cross-talk with other phytohormones (Broekgaarden et al., 2015; Lorenzo et al., 2003; Onkokesung et al., 2010; Adie et al., 2007). The differential induction of ACC synthase (ACS) and ACC oxidase (ACO) suggests distinct temporal dynamics between resistant and susceptible cultivars. Both cultivars showed rapid induction of ACO after the challenge, but only the resistant cultivar showed a sustained upregulation of ethylene biosynthesis into the late response through the induction of both ACO and ACS. Ethylene produced at wound sites enhances the plant’s sensitivity to JA, and with the induction of multiple ethylene-response factors (ERFs) including ERF1, in the late resistant response, the convergence between ET and JA signalling during herbivory is highlighted.

In contrast, salicylic acid (SA)-mediated responses appeared more prominent in the susceptible cultivar. SA signalling is classically associated with defence against biotrophic pathogens and piercing sucking insects that cause minimal tissue damage, such as aphids (Ali et al., 2024). The biosynthetic route for SA biosynthesis varies across plant species. In *Arabidopsis thaliana* and barley, biosynthesis is primarily dependent on the isochorismate synthase (ICS) pathway (Garcion et al., 2008; Hao et al., 2018) whereas in tobacco, rice and maize, SA is synthesized from benzoic acid in the phenylpropanoid pathway (Ogawa et al., 2006; Yuan et al., 2019; Zhu et al., 2025). In maize, phenylalanine ammonia-lyase (PAL) genes are essential for SA accumulation as they drive the production of benzoic acid (Yuan et al., 2019). In our study, PAL genes were strongly upregulated only in the susceptible cultivar, suggesting increased transcription of genes in the phenylpropanoid-derived SA biosynthetic pathway. In addition, SA-responsive PR genes including PR-1, PR-2 and PR-5, were predominantly identified among the susceptible DEGs. Notably, PR-1, a widely recognised molecular marker of SA-mediated signalling and systemic acquired resistance (SAR) was a prominent component of the susceptible response.

The susceptible cultivar’s delayed induction of possible JA producing 9-LOXs combined with activation of the PAL-mediated SA pathway, supports the interpretation that the susceptible cultivar showed a relatively stronger SA-associated and delayed JA-associated transcriptional signal. This interpretation is further supported by the induction of a possible WRKY53 in the late susceptible response. WRKY53 is known to promote SA-dominant signalling and suppress JA-mediated herbivore defences (Negi and Khurana, 2021; Hu et al., 2015; 2016; Zhang et al., 2020). Its induction in the susceptible cultivar may therefore have reinforced SA signalling downstream of PAL activation in the phenylpropanoid pathway, which could potentially influence the balance between SA- and JA/ET-associated transcriptional response, potentially contributing to a more compatible interaction with eldana.

Overall, these results indicate that sugarcane mounts a multifaceted defence response to eldana that integrates direct chemical defences, cyanogenic metabolism, hormone mediated immune priming, and indirect volatile-mediated responses. Differences in the timing, magnitude, and basal activation of these pathways between resistant and susceptible cultivars likely contribute to observed variation in resistance phenotypes. Resistance appears to be associated with a stronger or earlier JA- and ET- associated transcriptional response, whilst the susceptible cultivar tends to display a comparatively stronger SA-associated transcriptional profile.

### Structural reinforcement of the cell wall as a defence response to eldana

The plant cell wall is a dynamic structure that not only provides support but plays an important role in plant defence. Besides acting as a physical barrier, the plant cell wall stores defence related compounds that may be released during damage, degradation or wounding. Its defensive properties are determined by the composition and organization of a complex matrix of cellulose, polysaccharides, lignins and other polymers linked by structural and chemical modifications.

The biosynthetic pathways of secondary cell wall (SCW) components are highly co-regulated (Brown et al., 2005) and are orchestrated by a hierarchical transcriptional network of NAC and MYB transcription factors. NAC transcription factors function as primary regulatory switches (Mitsuda et al., 2007; Zhong et al., 2006), while MYB transcription factors act downstream to fine-tune pathway-specific gene expression (Zhong et al., 2007; McCarthy et al., 2009). The early induction of genes encoding NAC transcription factors in the resistant cultivar suggests that the regulatory cascade governing SCW formation is activated more rapidly in resistant plants, enabling earlier deployment of wall-based defences. The induction of MYB transcription factors induced in both cultivars likely reflect a shared downstream response, although this response appears more sustained in the resistant genotypes.

Increased expression of cellulose synthase (CESA) complexes and sucrose synthase genes indicates enhanced carbon allocation towards cellulose production during the late response to herbivory. In SCWs, cellulose provides the primary structural scaffold, whereas lignin functions as the reinforcing matrix polymer. The induction of cellulose biosynthesis was accompanied by increased expression of many enzymes in the phenylpropanoid pathway, including PAL, C4H, 4CL, HCT, CCR, CCoAOMT, and CSE, as well as oxidative polymerisation enzymes such as laccases and peroxidases. The upregulation of all these enzymes are consistent with active lignification at wound sites. Lignin deposition increases cell wall hardness, reduces digestibility (Buxton and Redfearn, 1997; Hanley et al., 2007), and forms an effective physical and chemical barrier that limits further insect feeding while restricting invasion by secondary pathogens (Vance et al., 1980; Nicholson and Hammerschmidt, 1992; Santiago et al., 2013).

Beyond cellulose synthesis and lignification, modification of the SCW matrix was also evident in the resistant response. The induction of xylan biosynthesis and xylan-modifying genes further supports coordinated secondary wall reinforcement, as xylan helps tether cellulose microfibrils and anchor lignin within the wall matrix (Scheller and Ulvskov, 2010; Kang et al., 2019). Defects in xylan synthesis compromise cell wall integrity (Brown et al., 2007; Crowe et al., 2021), and the upregulation of IRX and GUX family members in the resistant cultivar suggests a mechanically strengthened barrier in response to herbivory. Additional evidence for post-synthetic wall remodelling of the cellulose-xyloglucan network was provided by the induction of xyloglucan endotransglycosylase/hydrolase (XTH) genes in the resistant response. In celery, XTHs have been implicated in deterring phloem-feeding insects from settling (Divol et al., 2007). In parallel with wall reinforcement, genes involved in cell wall loosening and expansion, such as expansins, were suppressed in the late resistant response. This repression in genes that control cell wall extensibility suggests a shift in resource allocation away from growth and towards defence.

Collectively, these findings support a model in which sugarcane cultivars respond to eldana by rapidly activating SCW biosynthesis and lignification, and cell wall remodelling pathways consistent with potential fortification of stem tissues against further penetration and feeding. This structural defence appears to operate alongside the chemical and signalling-based defences identified above and may represent an important component of the resistant cultivars transcriptional response.

### Identification of presence absence variation region containing defence induced dirigent-like gene

A dirigent-like gene was identified that was strongly induced by eldana herbivory exclusively in the resistant cultivar. There was no evidence of expression in the susceptible cultivar and further investigations ascertained that this gene, identified as *ShDIR31* (Nobile et al., 2017), was absent from the genome of the susceptible cultivar. The identification of a defence-induced gene that is both transcriptionally responsive and genomically absent in the susceptible genotype provides evidence for a link between inducible defence-related expression, PAV, and genotype-specific resistance. PAV regions are often enriched for adaptive, lineage-specific genes involved in biotic stress responses and are not part of the core genome shared across cultivars.

Dirigent proteins are best known for their role in controlling the stereoselective coupling of monolignols during lignan biosynthesis (Davin et al., 1997; Paniagua et al., 2017). While lignin polymerisation itself is largely non-stereospecific, dirigent-mediated coupling of monolignols is essential for the production of lignans (Halls and Lewis, 2002), a class of phenolic compounds with well-established antioxidant, antimicrobial, antifungal, and anti-insect properties (Li et al., 2017; Harmatha and Dinan, 2003; Cho et al., 2007). The induction of a DIR-like gene in the resistant cultivar therefore suggests that, in addition to enhanced lignification, resistant plants may also upregulate genes associated with lignan-related chemical defences at sites of wounding and larval feeding.

Similar wound- and insect-induced expression of DIR and DIR-like genes has been reported, in other systems, particularly in response to stem-boring insects where rapid induction of DIR transcripts occurs in damaged stem tissues, supporting a conserved role for this protein family in herbivore defence (Ralph et al., 2006, Ralph et al., 2007; Paniagua et al., 2017).

The association analyses provided evidence that the ShDIR31 marker is significantly associated with reduced eldana damage in the genotypes evaluated in the late-stage selection pot trial. Marker presence substantially reduces the likelihood of susceptibility and is associated with lower PID damage.

From a breeding perspective, the association observed between the ShDIR31 marker and reduced internode damage is consistent with the quantitative nature of eldana resistance, where multiple loci and environmental factors are likely to contribute to phenotypic expression. Marker present genotypes showed a favourable shift towards lower PID values and a reduced frequency of susceptible classifications.

Given its significant association and biological relevance, *ShDIR31* represents a promising candidate marker for marker-assisted selection (MAS) pipelines. While integration with additional markers may further enhance predictive accuracy, the current results demonstrate that the *ShDIR31* PAV locus provides a potentially useful and reliable marker for enriching resistant genotypes and accelerating genetic gain in breeding programmes.

## Conclusion

This study demonstrates the power of RNA-seq based transcriptomics to unravel the complex defence responses activated in sugarcane during herbivory by *Eldana saccharina*. By comparing two cultivars with contrasting resistance phenotypes, we identified distinct defence strategies that not only differed in timing and magnitude, but also in the underlying regulatory networks. The resistant cultivar mounted a rapid and coordinated response involving jasmonic acid and ethylene signalling, accompanied by extensive reinforcement of the secondary cell wall. In contrast, the susceptible cultivar engaged a more salicylic acid-driven approach, characterised by strong induction of PAL and multiple SA-responsive PR genes. Both cultivars showed substantial overlap in the induction of chemical defences, including cyanogenic and protease inhibitors, and secondary metabolite biosynthesis. However, it appears to be the SA-biased response in the susceptible cultivar that may be associated with a less effective defence against chewing herbivores.

The combination of differential expression, co-expression clustering, and functional annotation was used to reveal key defence modules. These results advance our understanding of the molecular basis of insect resistance in sugarcane, providing practical avenues for applied research. Notably, this study uncovered the presence of *ShDIR31*, a defence induced dirigent-like gene within a presence/absence variation (PAV) region in the resistant genotype. When evaluated in a larger cohort of pre-release breeding genotypes, marker-present genotypes showed significantly reduced eldana damage and an association with eldana resistance. PAV-associated loci represent promising targets for development of molecular markers to accelerate breeding for eldana resistance, especially in a crop where complex polyploidy has hindered genomic progress. As the sugar industry prioritises reduced pesticide reliance and integrated pest management practices, cultivar-based resistance provides a sustainable, long-term solution for mitigating eldana damage.

## Data Availability

The datasets presented in this study can be found in online repositories. The names of the repository/repositories and accession number(s) can be found in the article/**Supplementary Material**.
